# Prescription practice and clinical utility of chest radiographs in a pediatric intensive care unit: a prospective observational study

**DOI:** 10.1186/s12880-021-00576-6

**Published:** 2021-03-09

**Authors:** Rajeev Gupta, Karthi Nallasamy, Vijai Williams, Akshay Kumar Saxena, Muralidharan Jayashree

**Affiliations:** 1 Pediatric Emergency and Intensive Care Units, Department of Pediatrics, Advanced Pediatrics Centre, Postgraduate Institute of Medical Education and Research (PGIMER), Sector-12, Chandigarh, 160012 India; 2 Department of Radiodiagnosis, Postgraduate Institute of Medical Education and Research (PGIMER), Chandigarh, India

**Keywords:** PICU, Chest radiograph, X-ray, On demand, Quality improvement, Ventilation

## Abstract

**Background:**

Chest radiograph (CXR) prescribing pattern and practice vary widely among pediatric intensive care units (PICU). ‘On demand’ approach is increasingly recommended as against daily ‘routine’ CXRs; however, the real-world practice is largely unknown.

**Methods:**

This was a prospective observational study performed in children younger than 12 years admitted to PICU of a tertiary care teaching hospital in India. Data were collected on all consecutive CXRs performed between December 2016 and April 2017. The primary outcome was to assess the factors that were associated with higher chest radiograph prescriptions in PICU. Secondary outcomes were to study the indications, association with mechanical ventilation, image quality and avoidable radiation exposure.

**Results:**

Of 303 children admitted during the study period, 159 underwent a total of 524 CXRs in PICU. Median (IQR) age of the study cohort was 2 (0.6–5) years. More than two thirds [n = 115, 72.3%] were mechanically ventilated. Most CXRs (n = 449, 85.7%) were performed on mechanically ventilated patients, amounting to a median (IQR) of 3 (2–5) radiographs per ventilated patient. With increasing duration of ventilation, the number of CXRs proportionately increased in the first two weeks of mechanical ventilation. In non-ventilated children, about two thirds (68%) underwent only one CXR. Majority of the prescriptions were on demand (n = 461, 88%). Most common indications were peri-procedure prescriptions (37%) followed by evaluation for respiratory disease status (24%). About 40% CXRs resulted in interventions; adjustment in ventilator settings (13.5%) was the most frequent intervention. In 26% (n = 138) of radiographs, image quality required improvement. One or more additional body part exposure other than chest and upper abdomen were noted 336 (64%) images. Children with > 3 CXR had higher PRISM III score, more often mechanically ventilated, had higher number of indwelling devices [mean (SD) 2.6 (1.2) vs. 1.7 (1.0)] and stayed longer in PICU [median (IQR) 11(7.5–18.5) vs. 6 (3–9)].

**Conclusion:**

On demand prescription was the prevalent practice in our PICU. Most non-ventilated children underwent only one CXR while duration of PICU stay and the number of devices determined the number of CXRs in mechanically ventilated children. Quality improvement strategies should concentrate on the process of acquisition of images and limiting the radiation exposure to unwanted body parts.

## Background

Chest radiograph (CXR) is one of the most requested investigation in Intensive Care Units (ICU). Even in the era of modern non-radiation imaging techniques usefulness of a bedside portable CXR has remained unchanged [1]. With advent of digital radiography, images can be quickly captured, read, easily saved, retrieved, distributed and presented in a Picture Archiving Communication System [2]. Daily morning routine CXR prescription was the standard practice in ICU particularly in those receiving invasive mechanical ventilation (IMV), under the assumption that daily imaging can diagnose or identify undetected complications early [3]. However, recent evidence suggests that daily CXR in ICU patients receiving IMV are of low diagnostic yield and have negligible impact on management decisions and patient centered outcomes [4–8]. The Choosing Wisely campaign (2012) recommended not to use diagnostic tests including CXR on daily basis as they added no benefit to patient care besides increasing cost and potential harm [9–11]. Currently, though most intensivists prefer an on-demand strategy, a wide variation in practice has been reported across hospitals caring for adults and at some places, the daily routine CXR is still common [12–15].

Little is known about the CXR prescribing pattern and practice in Pediatric intensive care units (PICU). An important concern with respect to daily CXR, particularly in children is unwanted or avoidable radiation. For any given effective radiographic imaging, children get 3 to 4 times higher radiation dose as compared to adults; younger children get considerably more radiation in comparison to older counterparts [16]. In addition, frequent radiographic procedures could add to the risk of device (endotracheal tube, central venous catheters) displacement in understaffed units particularly common in a low middle income (LMIC) setting. Given this premise, we felt that a prospective evaluation of indications, prescribing patterns and clinical utility of portable chest radiographs performed in PICU could help in identifying potentially avoidable CXR and eventually pave way for quality improvement, cost savings and reduction in unwanted radiation in children.

## Methods

This was a prospective observational study conducted in a tertiary PICU at a teaching and referral hospital from north India between December 2016 and April 2017. All children aged 12 years or younger admitted to PICU for whom a CXR was ordered were included. Children admitted to PICU in whom a CXR was performed outside the PICU premises [during Emergency Department (ED) stay or after surgical procedures in an X ray-suite] were excluded. Our PICU is an independent 15-bed medical unit, admitting about 900 children annually, and about half being mechanically ventilated. The consultants, pediatric intensive care fellows and postgraduates work in a twice daily rotation with availability of round the clock portable CXR facility. The investigator did not interfere with the imaging process, interpretation or any other aspect of clinical care. No additional imaging was performed as a part of this study. The study was approved by Institute Ethics Committee and a waiver of consent was granted.

### Data collection

The study investigator (RG) prospectively collected information on all consecutive portable CXR prescribed from PICU during the study period. Data were recorded in a pre-designed proforma containing details regarding primary indication, clinical examination findings that prompted the request, prescribing physician’s designation, timing of request, findings reported in CXR, quality and exposure of the images and interventions that were guided by CXR findings. Information regarding patient age, clinical diagnosis, type of respiratory support, duration of mechanical ventilation, number of indwelling devices and hospital outcomes were recorded.

### Study definitions

The prescription strategy has been categorized as “routine” if CXR was performed in the morning hours between 5 and 8 AM without specifying clinical indication in the request form. “On demand” was labelled if CXR was performed with a specific indication such as to confirm a positive clinical examination finding or verifying position of invasive lines/devices. High CXR exposure was defined when more than 3 CXRs were performed in a child during PICU admission.

### Outcomes

The primary outcome was to assess the factors that were associated with higher CXR prescriptions during PICU stay. Secondary outcomes were to study the indications, nature of prescription, image quality and avoidable radiation exposure.

### Statistical analysis

Descriptive statistics were summarized with  mean (standard deviation) and median (interquartile range) for normal and skewed distribution respectively. Univariate statistical analysis was performed with t-tests (continuous variables), and Chi-square test or Fisher’s exact test (categorical variables). Multivariable logistic regression analysis was carried out to identify the predictors of higher CXR prescription. All tests were two tailed and p-value less than 0.05 was taken as significant. Data analysis was done using SPSS statistical software (version 20.0, IBM SPSS Statistics, USA).

## Results

Of 303 children admitted during the study period, 159 (52%) who underwent at least one CXR while in PICU were enrolled into the study. Majority (n = 137, 86%) were transferred from pediatric ED (Table 1). Median (IQR) age of the study population was 2 (0.6–5) years. Nearly a third (n = 46, 29%) were admitted with respiratory disorders; pneumonia and/or acute respiratory distress syndrome (ARDS), bronchiolitis and bronchial asthma were the most frequent diagnoses. Acute central nervous system (CNS) infections and status epilepticus were the second common group (N = 29, 18.2%). Neuromuscular weakness, cardiac disorders and systemic infections/sepsis comprised of about 7% of each. About three fourth of study subjects (n = 115, 72.3%) were mechanically ventilated and 2 (1.3%) children received non-invasive ventilation. About a quarter had unfavorable outcome; thirty-two (20%) children died and care was discontinued in another 10 (6%) children.

**Table 1:** Characteristics of study subjects

Parameters	n = 159
Source of admission, n (%)	
Emergency room	137 (86)
In-patient wards	14 (9)
Post-surgical	8 (5)
Duration of hospital stay before PICU admission in days, Median (IQR)	1 (0–1)
Age in months, Median (IQR)	2 (0.6–5)
Male, n (%)	84 (52.8)
PRISM III score mean (SD)	18.3 (8.1)
Diagnosis n (%)	
Pneumonia/ARDS	32 (20)
Empyema	4 (2.5)
Bronchiolitis/Bronchial asthma	8 (5)
Diffuse alveolar hemorrhage	2 (1.3)
Acute CNS infections (meningitis/encephalitis)	23 (14.5)
Status epilepticus	6 (3.8)
LGBS/myelitis/snake envenomation	11 (6.9)
Acute gastroenteritis	5 (3.1)
Scrub typhus/staphylococcal sepsis	12 (7.5)
Cardiac disorders (congenital/myocarditis)	11 (6.9)
Sepsis	7(4.4)
Others	38 (24)
Respiratory support	
Invasive mechanical ventilation	115 (72.3)
Non-invasive ventilation	02 (1.3)
Non-ventilated	42 (26.4)
Outcome	
Survived to discharge	117 (73.6)
Discontinued care	10 (6.3)
Death	32 (20)

During the study period of 116 calendar days, a total 524 CXRs were performed in 159 children, amounting to 4.5 CXR per PICU day. Majority of the radiographs (n = 449; 85.7%) were performed on mechanically ventilated patients. About one third (31%) of all patients underwent only one CXR during their PICU stay. In non-ventilated patients (n = 42, 26.4%), this proportion rose to two thirds, i.e., about 68% of non-ventilated patients underwent only one CXR. However, in mechanically ventilated children, majority (n = 75, 65%) had 3 or more images [median (IQR)—3(2–5) radiographs per ventilated patient] (Table 2).

**Table 2:** Details of CXRs with respect to invasive ventilation

	Total (n = 159)	Invasive ventilation (n = 115)	NIV & Non-ventilated (n = 44)
Total CXR performed n (%)	524	449 (85.7)	75 (14.3)
No. of CXR per PICU day Median (IQR)	4.0 (3–6)		
No. of CXR per patient			
Median (IQR)	3.0 (1–4)	3.0 (2–5)	1.0 (1–2)
Range	(1–18)	(1–18)	(1–8)
No. of CXRs per patient n (%)			
1 CXR	50 (31.4)	20 (17.4)	30 (68.2)
2 CXRs	27 (17)	20 (17.4)	7 (16)
3 CXRs	25 (15.7)	22 (19)	3 (6.8)
4 CXRs	22 (14)	20 (17.4)	2 (4.5)
5 CXRs	12 (7.5)	12 (10.4)	0
6–10 CXRs	19 (12)	17 (15)	2 (4.5)
> 10 CXRs	4 (2.5)	4 (3.5)	0

Most CXR prescriptions (n = 491, 94%) were ordered by pediatric critical care fellows. (Table 3) Very few additional requests (n = 14, 2.5%) were made during morning rounds by a consultant. We observed a predominant on demand prescription [n = 461 (88%)]. Among the on-demand requests, about half had elective indications while just over a quarter (n = 127, 28%) had urgent or emergent indications for performing CXR as denoted in the request form. Although we intended to record the timeline of prescription to acquisition of image to identify any time delay in obtaining portable radiograph, the data was incomplete and hence not presented here. Peri-procedural prescriptions formed the most common indication for ordering a CXR (n = 194, 37%), majority being after endotracheal intubation (n = 79, 15%), prior to extubation (n = 32, 6%) and post intercostal tube drainage (n = 36, 7%). Only 2.5% (n = 14) radiographs were performed for confirmation of position of central venous catheters and none were performed for confirming feeding tube position. The other frequent indications for a CXR were ascertaining the baseline pulmonary status (n = 125, 23.8%), confirmation of abnormal clinical examination findings (n = 86, 16%) and hypoxemic episodes (n = 57, 11%). About 40% of images resulted in interventions. Adjustment in ventilator setting was the most common intervention (n = 71, 13.5%) followed by repositioning of endotracheal tube and changes in supportive care.

**Table 3:** Details of chest radiograph prescription and indications

Characteristics	n (%)
CXR prescription ordered by	n = 524
Consultant	14 (2.5)
Fellow	491 (94)
Postgraduate Resident	11 (2)
Not specified	08 (1.5)
Nature of prescription	n = 524
Routine	63 (12)
On demand	461 (88)
Nature of indications in ‘on-demand’ prescriptions n (%)	n = 461 (88)
Elective indications	241 (52)
Urgent/emergent indications	127 (28)
Unclassified	93 (20)
Indications for CXR n (%)	n = 524
To evaluate the respiratory disease status	125 (23.8)
Acute reduction in unilateral air entry	86 (16.2)
New onset desaturation	57 (11)
Peri-procedure	194 (37)
Others	62 (12)
Peri-procedure indications n (%)	n = 194 (37)
Post-intubation	79 (41)
Pre-extubation	32 (16.5)
Post-extubation	17 (8.7)
Central venous catheter position assessment	14 (7)
Post intercostal tube insertion	36 (18.5)
Post intercostal tube removal	01 (0.5)
Confirmation of feeding tube position	0
Post tracheostomy	10 (5)
Post bronchoscopy	05 (2.5)
Interventions after CXR n(%)	n = 204 (39)
Endotracheal tube repositioning	51 (25)
Central Venous Line repositioning	02 (1)
Addition/change of antimicrobials	04 (2)
Adjustment in Ventilator setting	71(35)
Changes in supportive care (Physiotherapy, position change)	47 (23)
Intercostal tube drainage	06 (3)
Others	23 (11)

**Fig. 1:**
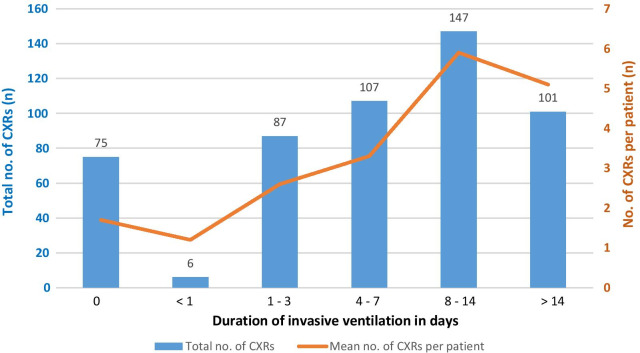
Relationship between duration of invasive ventilation and number of CXRs

With increasing duration of invasive ventilation, the mean number of CXRs per patient proportionately increased in the first two weeks (Fig. 1). Children who were ventilated for 4 to 7 days duration underwent an average of 3.3 CXRs as compared to 5.9 CXRs in those who received mechanical ventilation for 8 to 14 days. However, in children requiring ventilation beyond 2 weeks, the average no. of CXRs had decreased to 5.1 images. Details about the process of acquisition of CXR images and interpretation are shown in Table 4. The image findings were obtained from clinical records and radiologist’s reports and independently interpreted by investigators (RG & KN) for agreement. Of 524 images, 15% (n = 79) did not have any abnormal findings and were reported as normal. Unilateral/bilateral infiltrates, consolidation or collapse were the most common findings seen in 51% of images. Pneumothorax was noted in 6%. In 26% (n = 138) of radiographs, image quality required improvement. One or more additional body part exposure other than chest and upper abdomen were noted 336 (64%) images.

**Table 4:** Details on process of acquisition of chest radiographic images

	N = 524 (%)
Details	
Adequate quality images	334 (64)
Incomplete penetration (requiring digital adjustment)	52 (10)
Image quality requiring improvement	138 (26)
Rotation	97 (18.5)
Artifact (Lines and tubes obstructing the field)	17 (3.2)
Inadequate inclusion/poor lung volume	24 (4.6)
Field of view	
Field of view limited to chest and upper abdomen	188 (36)
One or more additional fields exposed	336 (64)
Exposed fields	
Upper neck/lower face	190 (36.3)
Full face	10 (1.9)
Forearm—single	17 (3.2)
Forearm—both	12 (2.3)
Hand—single	8 (1.5)
Hand—both	2 (0.4)
Lower abdomen	322 (61)
Pelvis	79 (15)
Thigh—single	3 (0.6)
Thigh—both	27 (5.2)
Findings	
Normal	79 (15)
Not reportable	33 (6)
Unilateral/bilateral consolidation and/or collapse	266 (51)
Interstitial infiltrates	58 (11)
Hyperinflation	11 (2)
Pneumothorax	31 (6)
Pleural effusion	42 (8)
Cardiomegaly with normal lung fields	4 (0.7)

### Multivariable analysis

We compared children who underwent 3 or less CXRs with those who had > 3 images during their PICU stay (Table 5). Children with > 3 images had higher Pediatric risk of mortality III (PRISM III) score, more often mechanically ventilated, had higher number of indwelling devices [mean (SD) 2.6 (1.2) vs. 1.7(1.0)] and stayed longer in PICU [Median (IQR) 11(7.5–18.5) vs. 6 (3–9)]. On multivariable analysis, PRISM III score, length of PICU stay and number of indwelling devices were independently associated with higher CXR prescriptions.

**Table 5:** Predictors of higher CXR prescriptions

Variables	≤ 3 CXRs (n = 102)	> 3 CXRs (n = 57)	p value	Multivariable analysis	
				P value	Odds ratio (95% CI)
Age in years	2 (0.3–5.2)	2 (0.7–5)	0.5		
Median (IQR)					
Diagnosis n (%)					
Respiratory diseases	40 (39)	31 (54)	0.06		
Neurological disorders	34 (33.3)	16 (28)	0.49		
PRISM III score mean (SD)	16.5 (7.6)	21.6 (8)	0.001	0.02	1.07 (1.0–1.1)
Mechanically ventilated n (%)	62 (61)	53 (93)	< 0.001	0.15	2.8 (0.7–11.7)
No of indwelling devices mean (SD)	1.7 (1.0)	2.6 (1.2)	< 0.001	0.006	1.9 (1.2–3.0)
Length of PICU stay median (IQR)	6 (3–9)	11(7.5–18.5)	0.001	0.03	1.04 (1.0–1.1)

## Discussion

In this prospective study we found that most CXRs ordered in PICU were in mechanically ventilated children. The number of images increased proportionately during the first two weeks of invasive ventilation. Severity of illness at admission, number of indwelling devices and length of PICU stay were predictive of higher number of CXRs in children admitted to PICU. Only about half (n = 159, 52%) of our PICU admissions underwent a chest radiograph imaging during PICU stay. This finding highlights two important dynamics in our PICU functioning. Firstly, all our study children were admitted in ED or ward for a median duration of about 24 h before transfer to PICU and it is likely that some of them had undergone a CXR immediately before PICU admission. Secondly, the prevalence of ‘on-demand’ prescription (88%) could be another reason for the lower frequency of CXR prescription in admitted patients. Several centres have reported a change in practice from routine CXRs to on-demand prescriptions over the past two decades [13–15] Although a prospective study conducted in multi-institutional PICUs suggested routine CXRs to be useful in a cohort of critically ill children, subsequent randomized controlled trials in adults and consensus opinions strongly supported the adoption of on-demand strategy to minimize the use of radiography in mechanically ventilated patients without compromising quality of care or safety [3, 17].

Our study cohort included critically ill children who were younger and smaller; the median (IQR) age and weight were 2 (0.6–5) years and 12.6 (8.4) kg respectively. This is similar to the report by Quasney et al., where the median weight of the study cohort was 9 kg (range 2–103 kg) and 55% of CXRs were performed in children weighing ≤ 10 kg [3]. This study also reported that the possibility of an intervention being performed based on routine CXR was higher in children weighing ≤ 10 kg. Additionally, presence of indwelling devices was positively associated with CXR prescription and intervention, a finding similar to ours. Nearly 90% of routine CXRs were performed on children who had one or more devices and presence of 2 devices increased the intervention rate from 19% to > 50% [OR (95% CI) 5.3(2.5–11.5)]. Of 524 CXRs performed in our study, most (85%) were done on mechanically ventilated patients. This is in contrast to the previous study where only 65% were obtained in patients on mechanical ventilation [3]. This difference could have been due to the ‘routine’ prescription strategy practiced in the latter report. More than two thirds of non-ventilated children in our cohort required only one CXR while the number of CXR increased proportionately with duration of ventilation in children receiving mechanical ventilation. Children who required invasive ventilation for 1 to 3 days and 4 to 7 days were prescribed an average of 2.6 and 3.3 CXRs during their PICU stay, respectively. This figure compares favorably with the rate of prescription in a multicenter study where the mean (95% CI) number of ‘on demand’ CXR per patient per day of mechanical ventilation was 0.75 (0.6–0.8) [17]. Mechanically ventilated children, with presence of one or more devices, are at higher risk of respiratory complications and more likely to have dynamic changes in pulmonary findings. Hence, CXRs are frequently ordered in this cohort of PICU patients despite an ‘on demand’ approach.

We found that peri-procedure prescription (37%) and evaluation of pulmonary status in respiratory diseases (17%) were the most common on-demand indications for performing a CXR in our PICU. This is comparable to other studies where CXRs were often obtained after endotracheal intubation, intercostal tube placement and central venous catheterization and for diagnostic evaluation of pneumonia, ARDS and pneumothorax [15, 17–19]. The indications for a CXR after intubation and central line placement are not considered ‘on-demand’ and not supported by literature [15, 20, 21]. Similarly, pre and post extubation radiographic images are not routinely recommended and need further review to decrease unnecessary images and radiation exposure. In our study, about 12% CXRs were performed on mechanically ventilated children without clear written indication prior to order. A careful study of these prescriptions could provide further opportunities to identify unnecessary x-rays that can be avoided in PICU. However, such analysis can be difficult in intensive care settings. Studies show that intensivists tend to assume higher clinical value of CXR than its reported efficacy [18]. The ability of a negative CXR to exclude complications probably has a clinical impact that is often hard to study [19].

An intervention was performed post imaging in 204 out of 524 radiographs (39%). Most frequent interventions included adjustments in the ventilator setting and institution of chest physical therapy. About 10% of CXRs resulted in repositioning of devices such as endotracheal tubes and central venous catheters. The list of interventions after CXR in our study was comparable to previous report by Quasney et al. in their multi PICU study, although the proportions were different [3]. In mechanically ventilated children ≤ 10 kg, about 20% of CXRs resulted in adjustment of ET tube while change in ventilator settings was performed after about 10% of images. Randomized trials in adults and in PICU did not demonstrate increased need of intervention in routine CXR group. Clec’h et al. in their randomised trial found restrictive use of CXRs in mechanically ventilated patients to have better diagnostic and therapeutic efficacies without affecting mortality [5] Similarly, another single centre randomised trial in PICU showed that daily routine CXRs was not associated with reduced length of stay or mortality [22]. In our cohort, the predominant indication for CXR was a respiratory cause while in most studies it was related to cardiovascular or post-operative cause.

Our results on details of acquisition of radiographs and quality of images provided some important insights into the process and identified areas for improvement. With the advent of portable digital CXR, rate of repeat radiographs came down significantly [23]. However, about a quarter of all images posed difficulty in interpretation of findings as they had axis rotation or presence of artifacts due to lines and tubes. Though portable CXRs in intensive care settings has its limitations, this process can be improved upon to avoid errors in interpretation and subjecting children to repeated radiation exposure. It was also noted that in about two thirds of CXRs, children had additional body part exposure other than chest and upper abdomen, a finding similar to previous reports [24]. It may be challenging to limit extra exposure in small children. An x-ray exposure chart was proposed by Knight et al. to optimize digital radiography in order to lower radiation dose and improve image quality [25, 26]. However, the authors acknowledged the prevailing gaps in the literature in assisting the development of such a chart for children. The efforts require involvement of multiple stakeholders, including radiographers, radiologists, equipment vendors and clinicians. The exposures would need optimization based on age, size and equipment combination.

### Strengths and limitations

Our study has several strengths. This is one of the first studies in PICU to report the prescription practice and the process of acquisition of images from LMIC setting. We measured the association between mechanical ventilation, number of devices and the prescription of CXRs in a sizeable sample. A few limitations need mention. We did not measure the alternative imaging modalities (eg. ultrasound) used during the study period that could potentially replace CXR in certain circumstances. An interventional study could have been a more useful methodology in quantifying potential areas of quality improvement.

## Conclusion

On demand prescription for chest radiograph was the prevalent practice in our PICU. Most non-ventilated children underwent only one CXR while the length of PICU stay and the number of devices determined the number of CXRs in mechanically ventilated children. Quality improvement strategies should concentrate on the process of acquisition of images and limiting the radiation exposure to unwanted body parts.

## Data Availability

All data generated or analysed during this study are included in this published article. The dataset/radiographic images are available from the corresponding author on reasonable request.

## Abbreviations

CXR: Chest radiograph
ICU: Intensive care unit
IMV: Invasive mechanical ventilation
PICU: Pediatric intensive care unit
ED: Emergency department
LMIC: Low middle income countries
ARDS: Acute respiratory distress syndrome
CNS: Central nervous system
PRISM III: Pediatric risk of mortality III
